# Toxin acidic residue evolutionary function-guided design of de novo peptide drugs for the immunotherapeutic target, the Kv1.3 channel

**DOI:** 10.1038/srep09881

**Published:** 2015-05-08

**Authors:** Zongyun Chen, Youtian Hu, Jing Hong, Jun Hu, Weishan Yang, Fang Xiang, Fan Yang, Zili Xie, Zhijian Cao, Wenxin Li, Donghai Lin, Yingliang Wu

**Affiliations:** 1 State Key Laboratory of Virology, College of Life Sciences, Wuhan University, Wuhan 430072, China; 2 College of Biological Science and Technology, Fuzhou University, Fuzhou 350108, China; 3 College of Chemistry and Chemical Engineering, Xiamen University, Xiamen 361005, China; 4 Center for BioDrug Research, Wuhan University, Wuhan 430072, China

## Abstract

During the long-term evolution of animal toxins acting on potassium channels, the acidic residues can orientate the toxin binding interfaces by adjusting the molecular polarity. Based on the evolutionary function of toxin acidic residues, de novo peptide drugs with distinct binding interfaces were designed for the immunotherapeutic target, the Kv1.3 channel. Using a natural basic toxin, BmKTX, as a template, which contains 2 acidic residues (Asp19 and Asp33), we engineered two new peptides BmKTX-19 with 1 acidic residue (Asp33), and BmKTX-196 with 2 acidic residues (Asp6 and Asp33) through only adjusting acidic residue distribution for reorientation of BmKTX binding interface. Pharmacological experiments indicated that BmKTX-19 and BmKTX-196 peptides were specific inhibitors of the Kv1.3 channel and effectively suppressed cytokine secretion. In addition to the structural similarity between the designed and native peptides, both experimental alanine-scanning mutagenesis and computational simulation further indicated that the binding interface of wild-type BmKTX was successfully reoriented in BmKTX-19 and BmKTX-196, which adopted distinct toxin surfaces as binding interfaces. Together, these findings indicate not only the promising prospect of BmKTX-19 and BmKTX-196 as drug candidates but also the desirable feasibility of the evolution-guided peptide drug design for discovering numerous peptide drugs for the Kv1.3 channel.

The voltage-gated Kv1.3 potassium channel is expressed in effector memory T cells and has been proven to be an attractive drug target for the treatment of various autoimmune diseases^1^,^2^. Kv1.3 channel blockers suppressed cytokine secretion and alleviated diseases in animal models of T cell-mediated autoimmune diseases^1^,^3^. Due to the inherent poor selectivity and potential side effects of previously reported chemical molecules targeting the Kv1.3 channel^4^,^5^, considerable attention has been paid to the discovery of peptide drugs recently.

During long-term molecular evolution, venoms from different species, such as scorpion, sea anemone, snake and cone snail, have become a well-known resource for peptide blockers that target the Kv1.3 channel^6^,^7^,^8^. To date, a large number of toxin peptides have been shown to inhibit the Kv1.3 channel at picomolar to nanomolar concentrations^9^. Although these peptides show better selectivity towards the Kv1.3 channel than chemical molecules, they usually also inhibit some highly similar potassium channel subtypes^9^,^10^,^11^,^12^. To further improve the selectivity of peptide candidates, some classical strategies were adopted, such as chemical modification of amino acid residues^13^, sequence truncation^14^, computer-aided design^10^ and phage display libraries^15^. Recently, the ShK-186 peptide, an analog of the anemone toxin peptide ShK, was identified as the first drug molecule to begin first-in-man phase-1 trials^11^,^16^. Despite the fact that the ShK-186 peptide blocks the Kv1.1 and Kv1.2 channels at nanomolar concentrations, clinical trial progress has greatly promoted the more extensive development of potent and selective Kv1.3 channel immunomodulators.

In this work, a new evolution-guided drug design strategy was proposed based on the evolutionary function of toxin acidic residues, which can orient the toxin binding interfaces by adjusting the molecular polarity, which was illustrated in Fig. 1. During the dominant electrostatic interactions between the positively charged binding interfaces of basic toxins and the negatively charged vestibule of the potassium channels^10^,^18^,^19^,^20^,^21^,^22^, these characteristically distributed acidic residues locate to the negatively charged non-binding interfaces of the basic toxins due to electrostatic repulsion forces between the acidic residues of both the toxins and potassium channels. Namely, toxin acidic residues can guide the orientation of toxin binding interfaces. This unique evolutionary role of toxin acidic residues has been elucidated for the highly similar toxins BmKTX, with 2 acidic residues (Asp19 and Asp33), and BmKTX-D33H and ADWX-1, each with 1 acidic residue (Asp19), which adopted distinct binding interfaces to recognize the Kv1.3 channel^10^,^22^ (Figs. 1B and 1C). Here, we applied a toxin evolution-guided strategy to design de novo peptide drugs using the natural toxin BmKTX as a template. By only adjusting the acidic residue distribution in the BmKTX template to orient BmKTX binding interface, two new peptides were designed: BmKTX-19 with 1 acidic residue (Asp33) and BmKTX-196 with 2 acidic residues (Asp6 and Asp33). Extensive experiments indicated that both designed peptides retained similar BmKTX structures but possessed two completely different binding interfaces from the BmKTX peptide. More significantly, the BmKTX-19 and BmKTX-196 peptides were identified as highly potent and selective blockers of the Kv1.3 channel. This work demonstrates that the BmKTX-19 and BMKTX-196 peptides are novel Kv1.3-specific drug candidates and also presents the promising prospect of designing peptide drugs utilizing the toxin evolution-guided strategy.

## Results

### Strategy for reorienting the binding interface of the Kv1.3 channel-blocking BmKTX peptide

Different types of basic toxins that target the Kv1.3 channel, such as ADWX-1, BmKTX, ShK and Kunitz-type peptides^10^,^20^,^22^,^23^,^24^, possess structural diversity at their binding interfaces. Therefore, it is feasible to reorientate the binding interface of the Kv1.3 channel toxin inhibitor to produce more promising drugs based on the evolutionary function of the toxin acidic residues.

**Figure 1:**
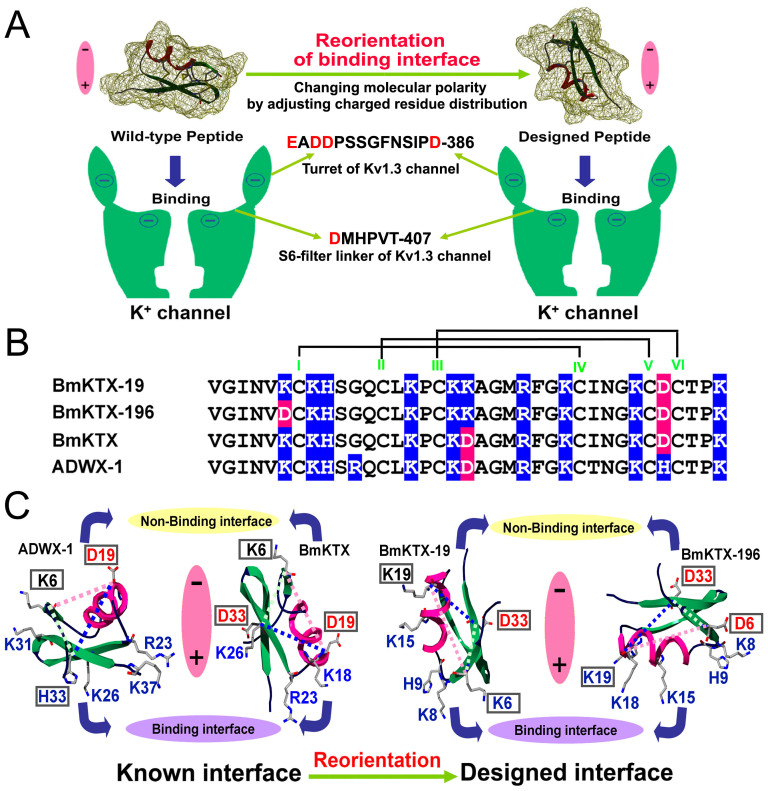
The strategy for reorienting the wild-type BmKTX binding interface to create two de novo peptides, BmKTX-19 and BmKTX-196, with potential new binding interfaces. A, Toxin evolution-guided drug design strategy and structural features of both peptide blockers and K^+^ channels. B, Sequence alignment of two designed and two known peptide blockers. Widely distributed basic residues are shaded in light blue, and characteristically distributed acidic residues are colored pink. C, Known binding interfaces of two potent Kv1.3 peptide inhibitors ADWX-1 (PDB code: 2K4U)^42^ and BmKTX (PDB code: 1BKT)^43^, and designed binding interfaces of two de novo BmKTX-19 and BmKTX-196 peptides. The basic residues around the peptide binding interfaces and acidic residues in the peptide non-binding interface were labeled. For the reorientation of the BmKTX binding interface by toxin evolution-guided drug design strategy, three key residues, Lys6, Asp19 and Asp33, in wild-type BmKTX and their corresponding residues in the designed BmKTX-19 and BmKTX-196 peptides were focused and framed.

To verify the feasibility of this strategy, we tried to reorientate the binding interface of the potent BmKTX peptide blocker using the turn motif between the α-helix and the anti-parallel β-sheet domains as its binding interface^22^. As shown in Figs. 1B and 1C, BmKTX and its potent analog ADWX-1 show different molecular polarities and adopt different molecular surfaces as their binding interfaces due to the strong electrostatic repulsion forces between the characteristically distributed acidic residues of the BmKTX and ADWX-1 peptides and the 20 total acidic residues of the tetrameric Kv1.3 channel^10^,^22^ (Fig. 1A). Based on these known binding interfaces, we further reoriented the binding interface of the BmKTX blocker to produce BmKTX-19 and BmKTX-196 blockers by changing the acidic residue distribution in the triangular vertices formed by Lys6, Asp19 and Asp33, whose synergetic effects would adjust the toxin molecular polarities and binding interfaces (Figs. 1B and 1C). In the designed BmKTX-19 blocker, the electrostatic repulsion forces between Asp19 of the BmKTX template and the acidic residues of Kv1.3 channel were removed, and the electrostatic repulsion forces between Asp33 of the BmKTX template and the acidic residues of Kv1.3 channel were preserved (Figs. 1B and 1C). Additionally, the introduction of Lys19 would not affect the molecular polarity because there are many basic residues in the BmKTX-19 peptide. This new distribution of acidic residues in BmKTX would likely rotate the BmKTX peptide by approximately 180°C and adopt a turn motif between the first β-sheet and α-helix domains to form the BmKTX-19 binding interface, which was opposite to that of wild-type BmKTX (Fig. 1C). In the designed BmKTX-196 blocker, the electrostatic repulsion forces between Asp19 of the BmKTX template and acidic residues of Kv1.3 channel were removed, and new electrostatic repulsion forces between toxin Asp6 and the acidic residues of the Kv1.3 channel were introduced. The new location of both Asp6 and Asp33 residues would likely rotate the BmKTX peptide by approximately 90°C and adopt the α-helix domain as the BmKTX-196 binding interface (Fig. 1C). Together, the regulated distributions of acidic residues in the BmKTX-19 and BmKTX-196 blockers resulted in unique molecular polarities, potentially allowing both blockers to adopt binding interfaces distinctly different from those of wild-type BmKTX and its analog, ADWX-1.

### BmKTX-19 and BmKTX-196 are potent and selective Kv1.3 channel blockers

**Figure 2:**
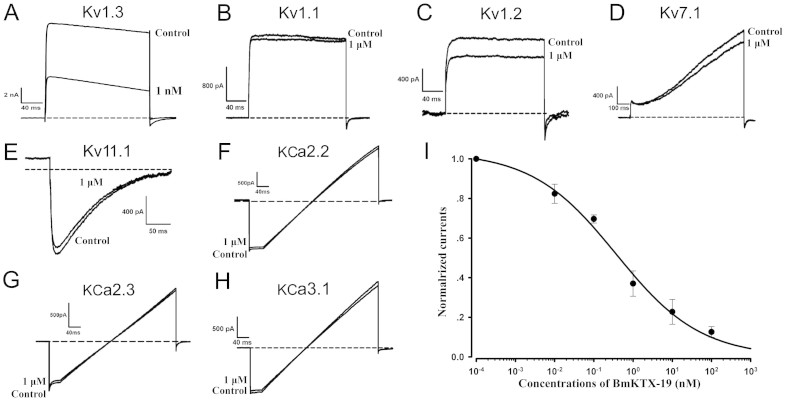
Pharmacological activities of BmKTX-19 on different potassium channels. A–H, Current traces in the absence (control) or presence of 1 nM BmKTX-19 on Kv1.3, and 1 μM BmKTX-19 on Kv1.1, Kv1.2, Kv7.1, Kv11.1, KCa2.2, KCa2.3 and KCa3.1 channels, respectively. I, Normalized current inhibition by various concentrations of BmKTX-19 on Kv1.3 channels. Data represent the means ± S.E. of at least three experiments.

**Figure 3:**
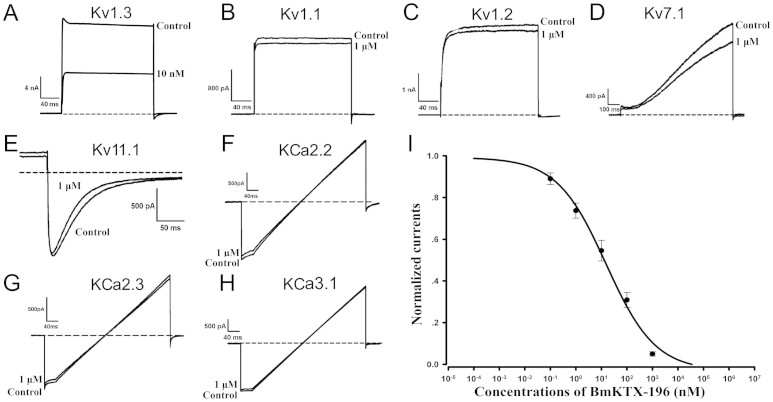
Pharmacological activities of BmKTX-196 on different potassium channels. A–H, Current traces in the absence (control) or presence of 10 nM BmKTX-196 on Kv1.3, and 1 μM BmKTX-196 on Kv1.1, Kv1.2, Kv7.1, Kv11.1, KCa2.2, KCa2.3 and KCa3.1 channels, respectively. I, Normalized current inhibition by various concentrations of BmKTX-196 on Kv1.3 channels. Data represent the means ± S.E. of at least three experiments.

To exploit the pharmacological profiles of the designed BmKTX-19 and BmKTX-196 peptide blockers, we produced recombinant BmKTX-19 and BmKTX-196 peptides using the previously reported procedures for preparing two similar recombinant peptides, ADWX-1 and BmKTX^10^,^22^ (supplementary Fig. S1). Then, we tested the pharmacological activities of the BmKTX-19 and BmKTX-196 peptides on Kv1.3 and other potassium channels. The results demonstrated that both peptides were potent Kv1.3 channel inhibitors. As shown in Figs. 2A and 3A, 1 nM BmKTX-19 and 10 nM BmKTX-196 inhibited more than 50% of Kv1.3 channel currents, which agreed well with the unusual sensitivity of the Kv1.3 channel towards different toxin peptide structures^22^,^23^,^25^. More interestingly, the BmKTX-19 and BmKTX-196 peptides showed much less of an effect on the channel currents of Kv1.1, Kv1.2, Kv7.1, Kv11.1, SKCa2, SKCa3 and IKCa at a much higher concentrations (1 μM) (Figs. 2B–2H and 3B–3H). The following concentration-response experiments indicated that the corresponding IC_50_ values of the designed peptides, BmKTX-19 and BmKTX-196, for inhibiting current were 0.375 ± 0.177 nM and 7.3 ± 1.8 nM, respectively (Figs. 2I and 3I). These data demonstrate that the BmKTX-19 and BmKTX-196 peptides, designed by the toxin evolution-guided strategy, are specific Kv1.3 channel blockers.

### The BmKTX-19 and BmKTX-196 blockers suppress cytokine secretion of T cells

The remarkable function of Kv1.3 channel peptide blockers is the ability to suppress cytokine secretion from human T cells by inhibiting Kv1.3 channel currents^7^,^15^,^26^. Based on the unique pharmacology of the designed BmKTX-19 and BmKTX-196 blockers, human T cells in PBMCs were used to confirm that both peptides displayed activity towards Kv1.3 channels in their natural milieu. As expected, both BmKTX-19 and BmKTX-196 were able to affect cytokine secretion when T cells in PBMCs were stimulated with anti-CD3/28 beads (supplementary Fig. S2). BmKTX-19 (10 nM) significantly inhibited the secretion of TNF-α, IL-2, and IFN-γ (supplementary Figs. S2A–S2C). However, BmKTX-196 markedly inhibited secretion of TNF-α, IL-2, and IFN-γ at higher concentrations (supplementary Fig. S2D–S2F). These differences in inhibiting cytokine production agreed with their differential Kv1.3 channel-blocking activities (Figs. 2 and 3).

### Structural similarity between the designed BmKTX-19 and BmKTX-196 peptides and natural BmKTX

**Figure 4:**
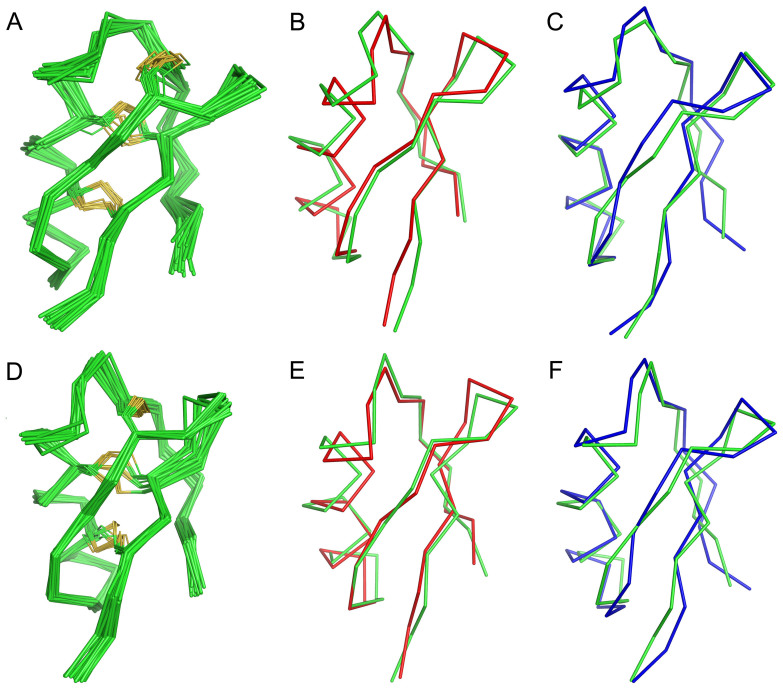
Solution structures of BmKTX-19 and BmKTX-196 peptide blockers. A, NMR-derived solution structure of BmKTX-19 (PDB code: 2MLA). The Cα traces (gray) of 20 structures of BmKTX-19 are superimposed. The side chains for cysteine residues and disulfide bonds are shown in yellow. B, Superimposition of the BmKTX-19 (green) structure with that of BmKTX (red, PDB code: 1 BKT). C, Superimposition of the BmKTX-19 (green) structure with that of ADWX-1 (blue, PDB code: 2K4U). D, NMR-derived solution structure of BmKTX-196 (PDB code: 2MLD. The Cα traces (gray) of 20 structures of BmKTX-196 are superimposed. The side chains for cysteine residues and disulfide bonds are shown in yellow. E, Superimposition of the BmKTX-196 (green) structure with that of BmKTX (red, PDB code: 1 BKT). F, Superimposition of the BmKTX-196 (green) structure with that of ADWX-1 (blue, PDB code: 2K4U).

Structural stability is essential for designing new toxin analogs using the toxin evolution-guided strategy. The favorable functions of the two designed BmKTX-19 and BmKTX-196 peptides prompted us to further investigate whether their structures remained similar to the wild-type BmKTX peptide after changing the acidic residue distribution. Thus, we used NMR spectroscopy to resolve the solution structures of the BmKTX-19 and BmKTX-196 blockers (supplementary Table S1 and S2, Fig. S3). The atomic coordinates were deposited in PDB (PDB codes: 2MLA,2MLD). The ensemble of the 20 lowest-energy structures of the BmKTX-19 and BmKTX-196 peptides was shown in Fig. 4. Overall, the mean structure of BmKTX-19 comprised a short α-helix (Leu14-Lys18) and a twist antiparallel β-sheet (Phe24-Cys27, Cys32-Thr35), whereas BmKTX-196 adopted almost the same fold but had a slightly longer helix (Ser10-Ala20) compared to BmKTX-19. The two structures were aligned with the structure of the wild-type BmKTX peptide, resulting in backbone root-mean-square-deviation values of 1.26 Å for BmKTX-19 and 1.25 Å for BmKTX-196 (Figs. 4B and 4E). Similarly, the pair-wise backbone root-mean-square deviations of the structure between the two peptides and ADWX-1, a BmKTX analog (Fig. 1), were 1.61 Å and 1.92 Å, respectively (Figs. 4C and 4F). The plot of the RMSD *versus* the residue number between BmKTX-19 and wild-type BmKTX or ADWX-1 shows that small RMSD regions are located in the secondary structure elements (supplementary Fig. S4A), while large RMDS regions are associated with the N-termini (V1-N4, C7-Q12). BmKTX-196 showed a similar pattern (supplementary Fig. S4B). These non-significant structural differences between the BmKTX-19, BmKTX-196, BmKTX and ADWX-1 peptides not only indicated that BmKTX-19 and BmKTX-196 adopted a similar structure as BmKTX to interact with the same target protein, the Kv1.3 channel but also revealed that the introduction of one or two acidic residues affected the molecular polarity of BmKTX-19 and BmKTX-196.

### Reorientation of the BmKTX binding interface in BmKTX-19 and BmKTX-196 blockers

**Figure 5:**
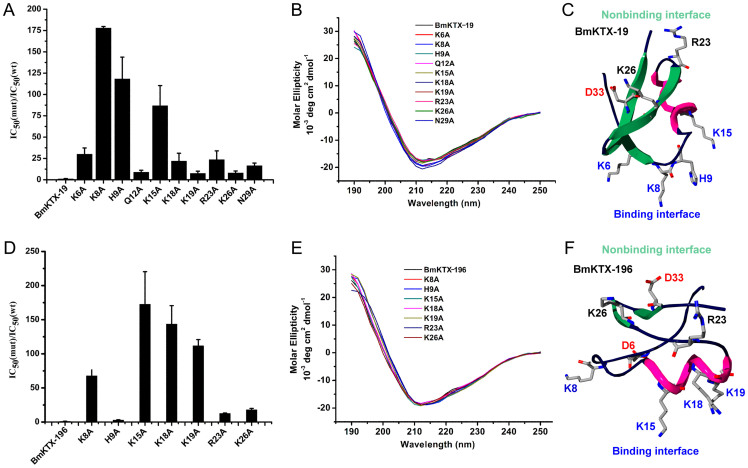
Binding interfaces and non-binding interfaces of BmKTX-19 and BmKTX-196 identified by alanine-scanning mutagenesis. A, Effects of BmKTX-19 and its alanyl mutants in blocking Kv1.3 channels. B, The circular dichroism spectra of recombinant peptides including BmKTX-19, BmKTX-19/K6A, BmKTX-19/K8A, BmKTX-19/H9A, BmKTX-19/K18A, BmKTX-19/K19A, BmKTX-19/R23A, BmKTX-19/K26A and BmKTX-19/N29A. C, Binding interface and non-binding interface of BmKTX-19; the key functional residues were marked blue. D, Effects of BmKTX-196 and its alanyl mutants in blocking Kv1.3 currents. E, The circular dichroism spectra of recombinant BmKTX-196, BmKTX-196/K8A, BmKTX-196/H9A, BmKTX-196/K15A, BmKTX-196/K18A, BmKTX-196/K19A, BmKTX-196/R23A, and BmKTX-196/K26A peptides. F, Binding interface and non-binding interface of BmKTX-196; the key functional residues were marked blue.

To verify whether the wild-type BmKTX binding interface was reoriented in the two de novo BmKTX-19 and BmKTX-196 blockers that were designed by the toxin evolution-guided strategy (Fig. 1), we individually measured the inhibitory effects of the peptide mutants on Kv1.3 activity using alanine-scanning mutagenesis. As expected, both the BmKTX-19 and BmKTX-196 peptides adopted binding interfaces that were distinctly different from wild-type BmKTX even though their structures were highly similar (Figs. 1, 4 and 5).

The pharmacological activity of the designed BmKTX-19 blocker, which contained only one aspartic acid residue (Asp33), relied on three essential residues (Lys8, His9 and Lys15) to interact with the Kv1.3 channel. Alanyl substitutions of these residues resulted in 178-, 118- and 87-fold decreases in affinity, respectively, compared to BmKTX-19 (Fig. 5A). Apart from alanyl substitutions of Lys8, His9 and Lys15, alanyl replacements at seven other positions (Lys6, Gln12, Lys18, Lys19, Arg23, Lys26 and Asn29) had much less of an impact on the inhibitory potency of BmKTX-19 (Fig. 5A). In combination with the similar CD spectra recorded for BmKTX-19 and all of its mutants (Fig. 5B), we identified the binding and non-binding interfaces for the BmKTX-19 blocker. As shown in Fig. 5C, the binding interface of the BmKTX-19 peptide was the turn motif between the first β-sheet and α-helix domains, in which the Lys8, His9 and Lys15 functional residues played important roles in peptide activity. The non-binding interface of BmKTX-19 was primarily formed by the terminal α-helix and the anti-parallel β-sheet domains, in which the non-essential residues, Lys18, Lys19, Arg23, Lys26 and Asn29, did not significantly affect peptide activity. The difference in binding interface orientation between the designed BmKTX-19 and wild-type BmKTX peptides indicated the essential function of acidic residues in adjusting molecular polarity and guiding the peptide inhibitor to bind to the Kv1.3 channel (Figs. 1B, 1C and 5C).

For the designed BmKTX-196 inhibitor, which contained two aspartic acid residues (Asp6 and Asp33), alanine-scanning mutagenesis experiments illustrated that single point mutations of four residues, Lys8, Lys15, Lys18 and Lys19, substantially reduced the affinity of BmKTX-196 for the Kv1.3 channel by 68-, 173-, 144-, and 112-fold, respectively (Fig. 5D). Alanine substitution of His9, Arg23 and Lys26 slightly altered BmKTX-196 inhibitory affinity towards Kv1.3 currents by 3-, 13-, and 18-fold, respectively. Together with the similar CD spectra of BmKTX-196 and all of its mutants (Fig. 5E), we easily identified the binding and non-binding interfaces of the BmKTX-196 inhibitor. As shown in Fig. 5F, BmKTX-196 primarily uses the α-helix domain as the binding interface, which contains three critical residues, Lys15, Lys18 and Lys19. Additionally, its non-binding interface is primarily located in the anti-parallel β-sheet domain containing the non-essential Arg23 and Lys26 residues. Distinct differences in the binding interface were observed for BmKTX-196, BmKTX-19 and wild-type BmKTX (Figs. 1C, 5C and 5F). These observations again highlight the function of acidic residues in orienting differential binding interfaces between Kv1.3 channel-blocking peptides.

## Discussion

Extensive attention has been paid recently to the discovery of peptide drugs targeting the Kv1.3 channel, an attractive target for autoimmune diseases^7^,^11^,^15^,^26^,^29^,^30^. In view of the diverse structural features of toxin peptide blockers from venomous animals^17^,^20^,^23^, we designed de novo peptide drugs based on the toxin acidic residue evolutionary function-guided strategy in the present work. Using a potent wild-type BmKTX inhibitor as a template, we obtained two de novo peptide blockers, BmKTX-19 and BmKTX-196, for the immunotherapeutic target, the Kv1.3 channel. The novel peptide inhibitors had the following remarkable features: (1) the BmKTX-19 and BmKTX-196 peptides are potent and selective blockers, with IC_50_ values of 0.375 ± 0.177 nM and 7.3 ± 1.8 nM, respectively (Figs. 1, 2 and 3); (2) the BmKTX-19 and BmKTX-196 peptides adopt a similar structure to the wild-type BmKTX template (Fig. 4); and more importantly, (3) the BmKTX-19 and BmKTX-196 peptides use the distinct binding interfaces to bind the Kv1.3 channel, which are completely different from those of wild-type BmKTX and its analog ADWX-1 peptide, although their amino acid sequences are almost identical (Figs. 1 and 5).

**Figure 6:**
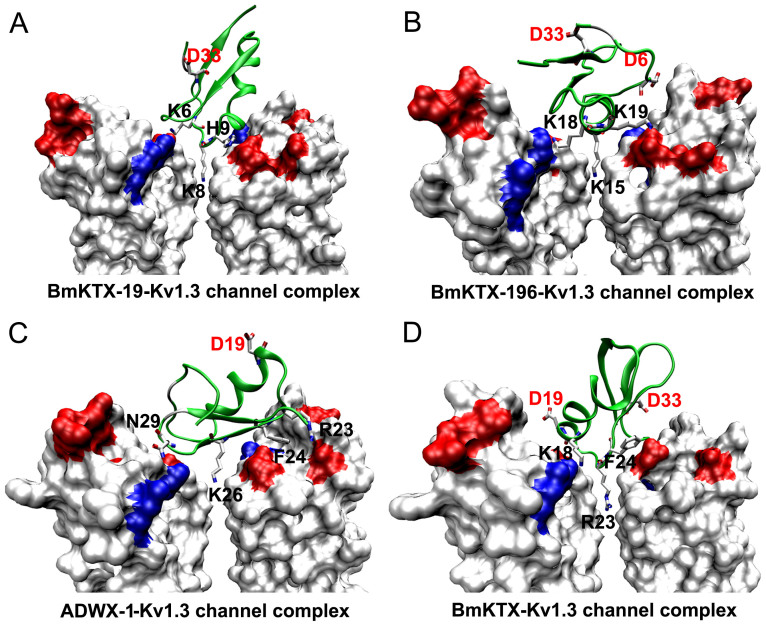
Distinct interaction modes between toxins and Kv1.3 channels mediated by the different distributions of toxin acidic residues. A, Interaction of BmKTX-19 with the Kv1.3 channel. B, Interaction of BmKTX-196 with the Kv1.3 channel. C, Interaction of ADWX-1 with the Kv1.3 channel, which is similar with the classical Lys26-blocking mode between BmKTX-D33H and Kv1.3 channel^10^,^22^. D, Interaction of BmKTX with the Kv1.3 channel^22^. Toxin acidic residues and key functional residues were marked.

To further illustrate the novel interactions of the two designed peptides, BmKTX-19 and BmKTX-196, towards the Kv1.3 channel, we constructed structural models of the peptide-Kv1.3 channel complexes for both peptides by computational simulations according to our previously reported methods^10^,^22^,^27^,^28^. In accord with our design strategy and experimental data (Figs. 1 and 5), the modeled complexes illustrate that the BmKTX-19 and BmKTX-196 inhibitors adopt distinct binding interfaces to recognize the Kv1.3 channel (Fig. 6 and supplementary Figs. S5 and S6). In the BmKTX-19 peptide-Kv1.3 channel complex, the BmKTX-19 turn motif between the first β-sheet and α-helix domains is used as the Kv1.3 channel-interacting interface, where three key residues, Lys8, His9 and Lys15, faced towards the channel pore region (Fig. 6A and supplementary Fig. S5A). While the most critical residue (Lys8) of the BmKTX-19 peptide was predicted to be the Kv1.3 channel pore-blocking residue (Figs. 5A and 6A, and supplementary Fig. S5B), structural analysis indicates that two other important residues His9 and Lys15 contact several residues of Kv1.3 channel through polar and non-polar interactions (supplementary Figs. S5C and S5D). In the BmKTX-196 peptide-Kv1.3 channel complex, the BmKTX-196 α-helix domain was identified as the Kv1.3 channel-interacting interface, where three key residues, Lys15, Lys18 and Lys19, faced towards the channel pore region (Fig. 6B and supplementary Fig. S6A). The most critical residue, Lys15, is located in the middle of the α-helix domain in the BmKTX-196 inhibitor and was predicted to be the Kv1.3 channel pore-blocking residue (Figs. 5D and 6B, and supplementary Fig. S6B). Other important residues located around the channel pore-blocking Lys15 residue, such as Lys18 and Lys19, were found to strongly interact with corresponding residues of Kv1.3 (supplementary Figs. S6C and S6D). These interactions of both BmKTX-19 and BmKTX-196 towards the Kv1.3 channels were significantly different from those of ADWX-1 (or BmKTX-D33H) with 1 acidic residue (Asp19) and the BmKTX template with 2 acidic residues (Asp19 and Asp33) (Figs. 6C and 6D)^10^,^22^. Through the electrostatic repulsion forces between toxin Asp19 and the negatively charged vestibule of the Kv1.3 channel, ADWX-1 or BmKTX-D33H mainly use Lys26 as the pore-blocking residue, and the antiparallel β-sheets as the interface to bind the Kv1.3 channel (Fig. 6C)^10^,^22^. However, the wild-type toxin BmKTX uses Arg23 as the pore-blocking residue, and uses the turn motif between the α-helix and antiparallel β-sheet domains to recognize Kv1.3 channel in the presence of toxin Asp19 and Asp33 residues (Fig. 6D)^22^. Although these predicted structural models may not always reveal the details of the actual interaction between toxins and Kv1.3 channels, these distinct interactions indicate that the BmKTX binding interface is, respectively reoriented in the two de novo BmKTX-19 and BmKTX-196 peptide inhibitors through the toxin acidic residue evolutionary function-guided strategy used in this work (Fig. 1).

In conclusion, two de novo BmKTX-19 and BmKTX-196 peptide inhibitors that target the Kv1.3 channel were designed using the toxin evolution-guided drug design strategy. While they use distinct binding interfaces compared to the BmKTX template, they both show desirable structural stability and high selectivity towards the immunotherapeutic target, the Kv1.3 channel. Our work not only indicates the promising prospect of Kv1.3 channel-selective drug candidates with the unique binding interfaces but also demonstrates that the toxin evolution-guided drug design strategy would fuel the peptide drug discovery pipeline in the near future.

## Methods

### Toxin site-directed mutagenesis

BmKTX-19 and BmKTX-196 peptides were generated by overlapping PCR and inserted into pGEX-6P-1 expression vector. The QuickChange Site-Directed Mutagenesis Kit (Stratagene, U.S.A.) was used to produce BmKTX-19 and BmKTX-196 mutants based on the wild-type pGEX-6P-1-BmKTX-19 and pGEX-6P-1-BmKTX-196 plasmids. All plasmids were verified by DNA sequencing before expression.

### Peptides and potassium channels

BmKTX-19 and BmKTX-196 peptides and their mutated analogs were produced according to a previously described procedure^10^,^22^. After the expression vector was transformed into *E. coli* Rosetta (DE3) cells, the cells were cultured at 37°C in LB medium with ampicillin (100 μg/mL). When the cell density reached an OD_600_ value of approximately 0.6, 0.5 mM isopropyl thio-b-D-galactoside (IPTG) was added to induce expression at 28°C. Cells were harvested after 4 hours and resuspended in 50 mM Tris-HCl/(pH 8.0)/10 mM Na_2_EDTA. Supernatant from the bacterial cell lysate was loaded to a GST-binding column. The purified fusion protein was then desalted using a centrifugal filter (Millipore, USA), and cleaved by enterokinase (Biowisdom, China) for 16 h at 25°C. Protein samples were then separated by HPLC on a C18 column (10 × 250 mm, 5 μm) (Elite-HPLC, China) using a linear gradient from 10% to 80% acetonitrile with 0.1% TFA for 60 min, and detected at 230 nm. The peptides were eluted from the major peaks at 20–25% acetonitrile. The molecular masses of the purified peptides were measured by MALDI-TOF-MS (Voyager-DESTR, Applied Biosystems). The cDNAs encoding mouse Kv1.1, human Kv1.2, mouse Kv1.3, human Kv7.1, human Kv11.1, human KCa3.1, human KCa2.2 and human KCa2.3 were subcloned into the *Xho* I and *Bam* HI sites of pIRES2-EGFP, a bicistronic expression vector (Clontech, USA) for coexpression with enhanced GFP.

### Circular Dichroism (CD) spectroscopy

Secondary structures for BmKTX-19, BmKTX-196 and their mutants were measured by Circular Dichroism (CD) spectroscopy. All samples were dissolved in water at a concentration of approximately 0.2 mg/ml. Spectra were recorded from 250 to 190 nm at 25°C with a scan rate of 50 nm/min, on a Jasco-810 spectropolarimeter (Jasco Analytical Instruments, Easton, MD, USA). The CD spectra were collected from the average of three scans after subtracting the blank spectrum for water.

### Cytokine secretion assay

For the cytokine secretion assay, 1 × 10^5^ freshly isolated PBMCs were activated using anti-CD3/CD28 Dynabeads (Invitrogen) at a cell:bead ratio of 1:1 in 200 μL of RPMI medium in 96-well plates. BmKTX-19 and BmKTX-196 were diluted at different concentrations in PBS and were added 1–2 hours prior to bead stimulation. All assays were performed in triplicate. After 16 hours of activation, the cells were counted and the supernatants were analyzed using ELISAs for IL-2, TNF-α and IFN-γ according to the manufacturer's instructions (eBiosciences).

### NMR experiments

NMR samples contained approximately 1–2 mM peptide in 500 L of 90% PBS/10% D_2_O at pH 4.5. All NMR experiments were carried out on a 600-MHz Bruker AV600 spectrometer equipped with three RF channels and a triple resonance cryoprobe. The 2D TOCSY spectra were acquired with a mixing time of 75 ms, and NOESY spectra were acquired with mixing times of 100 ms, 200 ms and 300 ms. Both the Watergate approach and the pre-saturation scheme were employed for water suppression. All spectra were recorded with 400 t1 increments and 2048 complex data points. Signals were averaged over 32 transients. All NMR data were processed and analyzed using NMRPipe/NMRDraw software and the Sparky program^31^,^32^. Linear prediction in the t1 dimension was used before the Fourier transformation.^1^H resonance assignments were performed using TOCSY, NOESY and COSY spectra to identify the scalar coupled spin systems and the sequential connectivity.

### Tertiary structure calculation

^1^H-1H distance restraints were derived primarily from the NOESY spectra, which were recorded in PBS with a mixing time of 100 ms. The structure calculations were performed according to the standard ARIA/CNS protocol^33^,^34^,^35^. NOE distance and J-coupling constraints were shown in Tables S1 and S2. A family of 200 structures was calculated according to the simulated annealing protocol and the 20 lowest-energy structures were finally selected. The root-mean-square deviation (RMSD) values for the backbone atoms of BmKTX-19 and BmKTX-196 are 0.48 Å and 0.44 Å, respectively, and were calculated by the program MOLMOL^36^. Ramachandran plot analysis was performed using the PROCHECK program^37^. The ribbon graphs were displayed by the software PyMol ((kindly provided by Prof. DeLano WL). The atomic coordinates of BmKTX-19 and BmKTX-196 have been deposited in the Protein Data Bank (PDB codes: 2MLA, 2MLD).

### Electrophysiological recordings

Electrophysiological experiments were carried out using patch-clamp whole-cell recording as described previously^10^,^23^,^27^,^38^. HEK293 cells were transfected with appropriate cDNA plasmids using the SofastTM Transfection Reagent (Sunma). Potassium currents were recorded 1 to 3 days after transfection and positive cells were selected based on the presence of GFP fluorescence. Electrophysiological experiments were carried out at 22–25°C using patch-clamp whole-cell recording. Data analyses were performed with IgorPro (WaveMetrics, Lake Oswego, OR), and IC_50_ values were deduced by fitting a modified Hill equation to the data, I_toxin_/I_control_ = 1/1 + ([toxin peptide]/IC_50_), where I is the peak current to the normalized data points obtained with at least five different toxin peptide concentrations. The results are mainly shown as the mean ± S.E, with n representing the number of experiments.

### Molecular simulations of peptide-Kv1.3 channel interactions

Molecular simulations were used to address the molecular mechanism of peptide-Kv1.3 channel interactions as we have described previously^22^,^27^,^28^. The 3D structures of the mouse Kv1.3 channel both free and in complex with either BmKTX-19 or BmKTX-D196 were modeled using the KcsA channel in its closed-state conformation (PDB code: 1BL8)^39^. The ZDOCK program was used to generate the candidate complex structures^40^. The interaction energies of wild-type BmKTX-19 and BmKTX-196 mutants with the Kv1.3 channel were calculated using the AMBER 8 package^41^.

## Author Contributions

The following authors read and approved the final manuscript: Z.Y.C., Y.T.H., J.H., J.H., W.S.Y., F.X., F.Y., Z.X., Z.J.C., W.X.L., D.H.L. and Y.L.W. The following authors conceived and designed the experiments: Y.L.W., D.H.L. and Z.Y.C. The following authors performed the experiments: Z.Y.C., Y.T.H., J.H., J.H., W.S.Y. and F.X. The following authors analyzed the data: Z.Y.C., Z.X., Z.J.C. and W.X.L. The following authors contributed reagents, materials, or analysis tools: F.X., F.Y. and Z.X. The following authors wrote the paper: Y.L.W., D.H.L., Z.Y.C. and J.H.

## Supplementary Material

Supplementary Information Supplementary informations
