# Supplementary material for: Toxin acidic residue evolutionary function-guided design of de novo peptide drugs for the immunotherapeutic target, the Kv1.3 channel
Source: Sci Rep. 2015 May 8;5:9881. doi: 10.1038/srep09881 (PMC4424837; doi:10.1038/srep09881)
Supplement: Supplementary Information — s [file srep09881-s1.doc]

**Supplementary informations**

**Toxin acidic residue evolutionary function-guided design of de novo peptide drugs for the immunotherapeutic target, the Kv1.3 channel**

**Zongyun Chen^#, 1^, Youtian Hu^#, 1^, Jing Hong^#, 2^, Jun Hu^1^, Weishan Yang^1^, Fang Xiang^1^, Fan Yang^1^, Zili Xie^1^, Zhijian Cao^1, 4^, Wenxin Li^1, 4^, Donghai Lin^*, 3^, Yingliang Wu^*, 1, 4^**

^1^State Key Laboratory of Virology, College of Life Sciences, Wuhan University, Wuhan 430072, China;

^2^College of Biological Science and Technology, Fuzhou University, Fuzhou 350108, China;

^3^College of Chemistry and Chemical Engineering, Xiamen University, Xiamen 361005, China;

^4^Center for BioDrug Research, Wuhan University, Wuhan 430072, China.

**^#^**These authors contributed equally to this work.

**^*^**Correspondence to: Donghai Lin and Yingliang Wu.

College of Chemistry and Chemical Engineering, Xiamen University, Xiamen 361005, China. E-mail: [dhlin@mail.shcnc.ac.cn](mailto:dhlin@mail.shcnc.ac.cn) (Donghai Lin); State Key Laboratory of Virology, College of Life Sciences, Wuhan University, Wuhan, China. Tel: ++86-27-68752831. Fax: ++86-27-68752146. E-mail: [ylwu@whu.edu.cn (Yingliang](mailto:ylwu@whu.edu.cn (Yingliang) Wu).

**Supplementary tables**

Table S1. Structure statistics of BmKTX-19 from the NMR structural analysis

| Quantity Value | | |
| --- | --- | --- |
| Total unambiguous distance restraints  Intra residual  Sequential ( | i – j | = 1)  Medium (2 ≤|I – j|≤ 4)  Long range ( | i – j | ≥ 5) | 480 | |
|  | 183 | |
|  | 149 | |
|  | 60 | |
|  | 88 | |
| Dihedral angle restraints | 22 | |
| Hydrogen bonds | 14 | |
| RMSD from the average atomic coordinates （1-37, Å） | | |
| Backbone atoms  All heavy atoms | 0.48 ± 0.08 | |
|  | 0.89 ± 0.10 | |
| Deviations from idealized covalent geometry | | |
| Bond (Å)  Angles (°)  Improper (°) | | 0.004 ± 0.0002 |
|  |  | 0.53 ± 0.02 |
|  |  | 1.52 ± 0.16 |
| Ramachandran analysis (%) | | |
| Residues in most favored regions  Residues in additional allowed regions  Residues in generously allowed regions  Residues in disallowed regions | 80.9% | |
|  | 18.8% | |
|  | 0.36% | |
|  | 0.0% | |

Table S2. Structure statistics of BmKTX-196 from the NMR structural analysis

| Quantity Value | | |
| --- | --- | --- |
| Total unambiguous distance restraints  Intra residual  Sequential ( | i – j | = 1)  Medium (2 ≤|I – j|≤ 4)  Long range ( | i – j | ≥ 5) | 452 | |
|  | 176 | |
|  | 145 | |
|  | 45 | |
|  | 86 | |
| Dihedral angle restraints | 21 | |
| Hydrogen bonds | 13 | |
| RMSD from the average atomic coordinates （1-37, Å） | | |
| Backbone atoms  All heavy atoms | 0.44 ± 0.09 | |
|  | 0.88 ± 0.09 | |
| Deviations from idealized covalent geometry | | |
| Bond (Å)  Angles (°)  Improper (°) | | 0.004 ± 0.0002 |
|  |  | 0.61 ± 0.03 |
|  |  | 1.99 ± 0.16 |
| Ramachandran analysis (%) | | |
| Residues in most favored regions  Residues in additional allowed regions  Residues in generously allowed regions  Residues in disallowed regions | 78.0% | |
|  | 21.4% | |
|  | 0.54% | |
|  | 0.0% | |


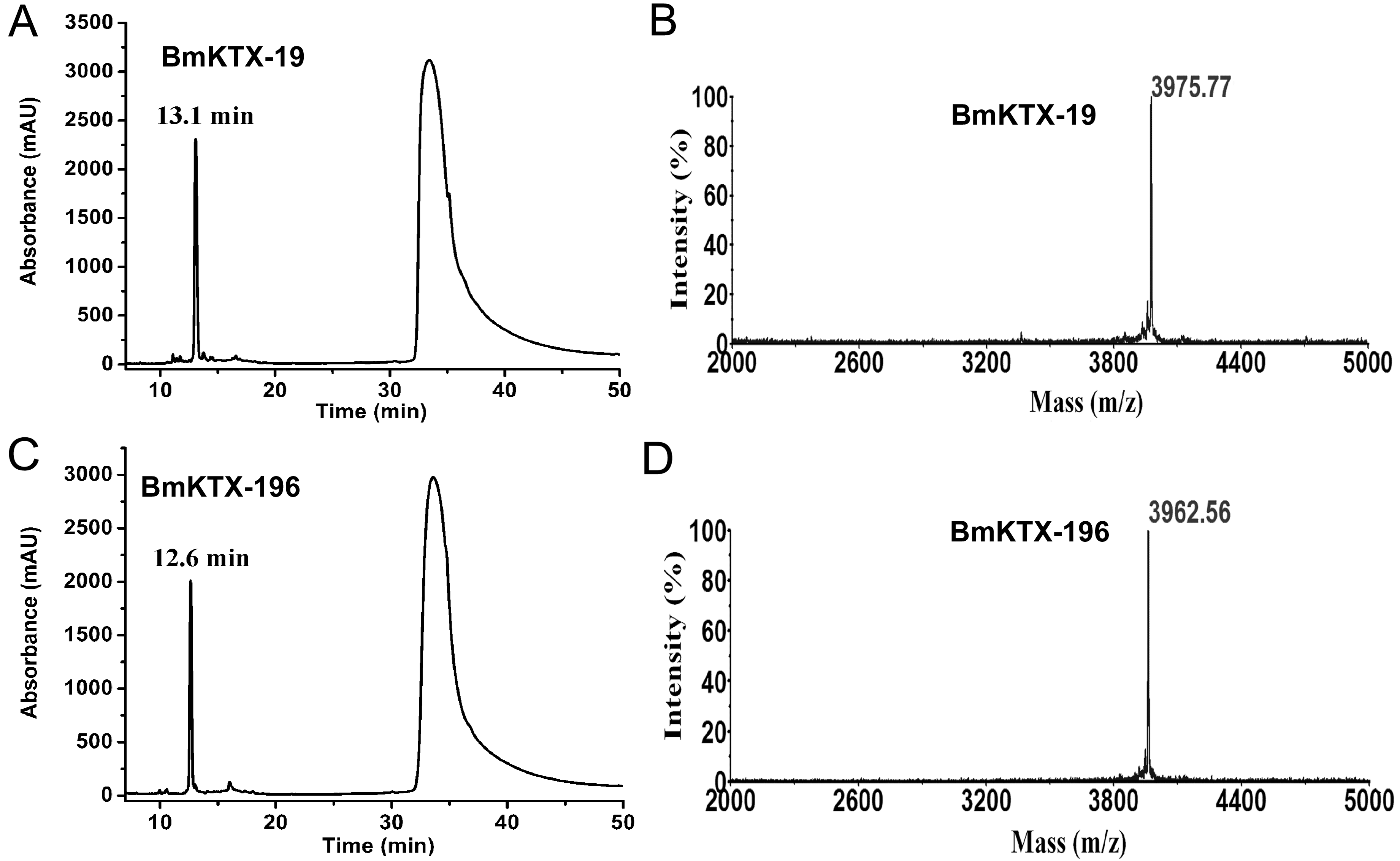
Figure S1. Purification and mass spectra of BmKTX-19 and BmKTX-196. A, Purification of BmKTX-19 by using an HPLC system equipped with a C18 reversed-phase column. B, Mass spectrum of BmKTX-19. The experimental mass value is 3975.77 Da (the deduced value is 3975.86 Da). C, Purification of BmKTX-196 by HPLC on a C18 column. The HPLC fractions that contain each peptide are indicated by their peak elution times. D, Mass spectrum of BmKTX-196. The experimental value is 3962.56 Da (the deduced value is 3962.78 Da).


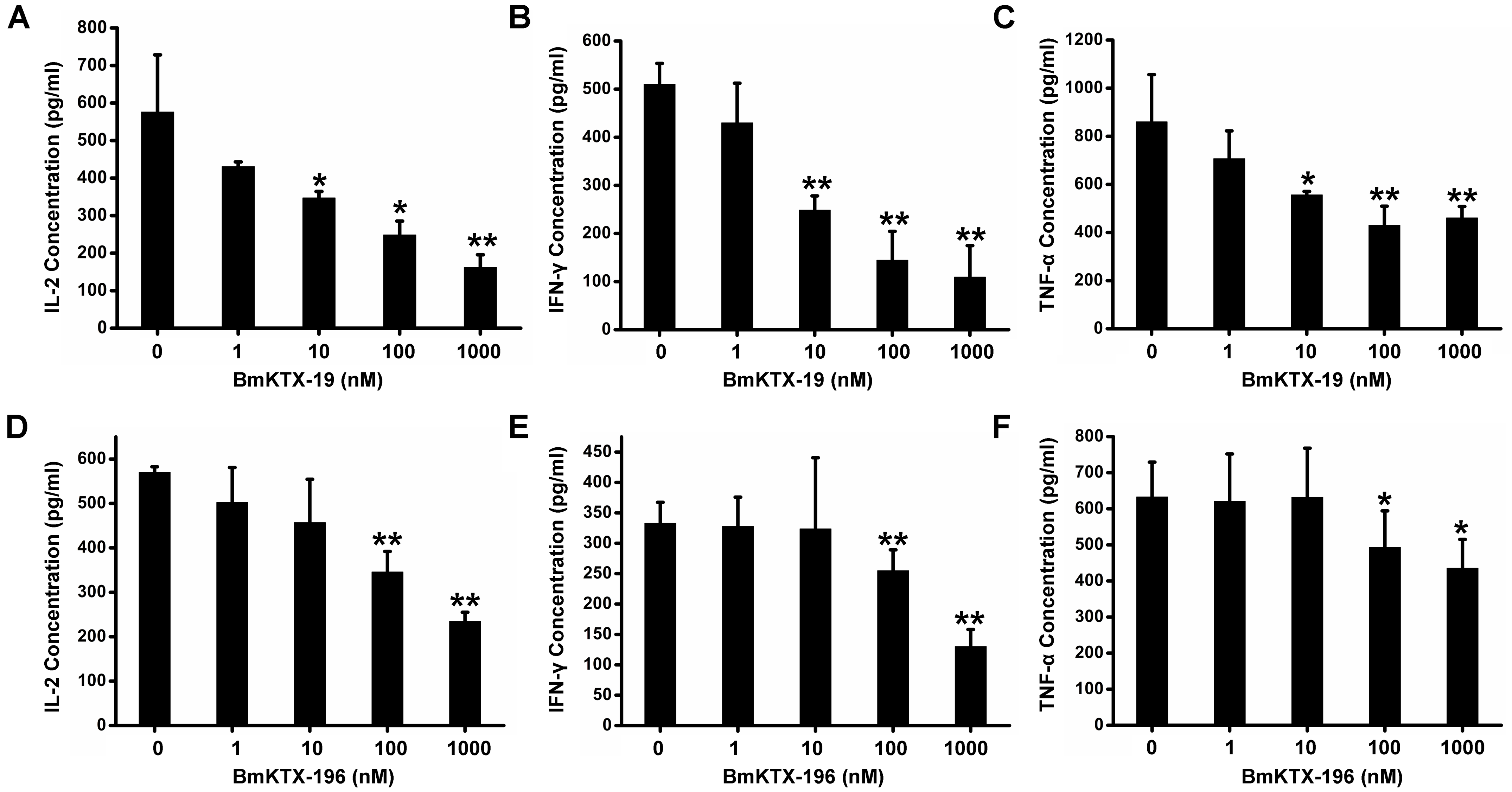
Figure S2. Two designed Kv1.3 peptide inhibitors inhibited cytokine production in T cells from PBMCs. A, BmKTX-19 suppressing IL-2 production in T cells from PBMCs. B, BmKTX-19 suppressing IFN-γ production in T cells from PBMCs. C, BmKTX-19 suppressing TNF-α production in T cells from PBMCs. D, BmKTX-196 suppressing IL-2 production in T cells from PBMCs. E, BmKTX-196 suppressing IFN-γ production in T cells from PBMCs. F, BmKTX-196 suppressing TNF-α production in T cells from PBMCs. The data shown are the mean ± SD from three independent experiments; statistically significant inhibitions compared with no drug treatment are determined using Student’s *t*-test. (**p*<0.05, ** *p*<0.01)


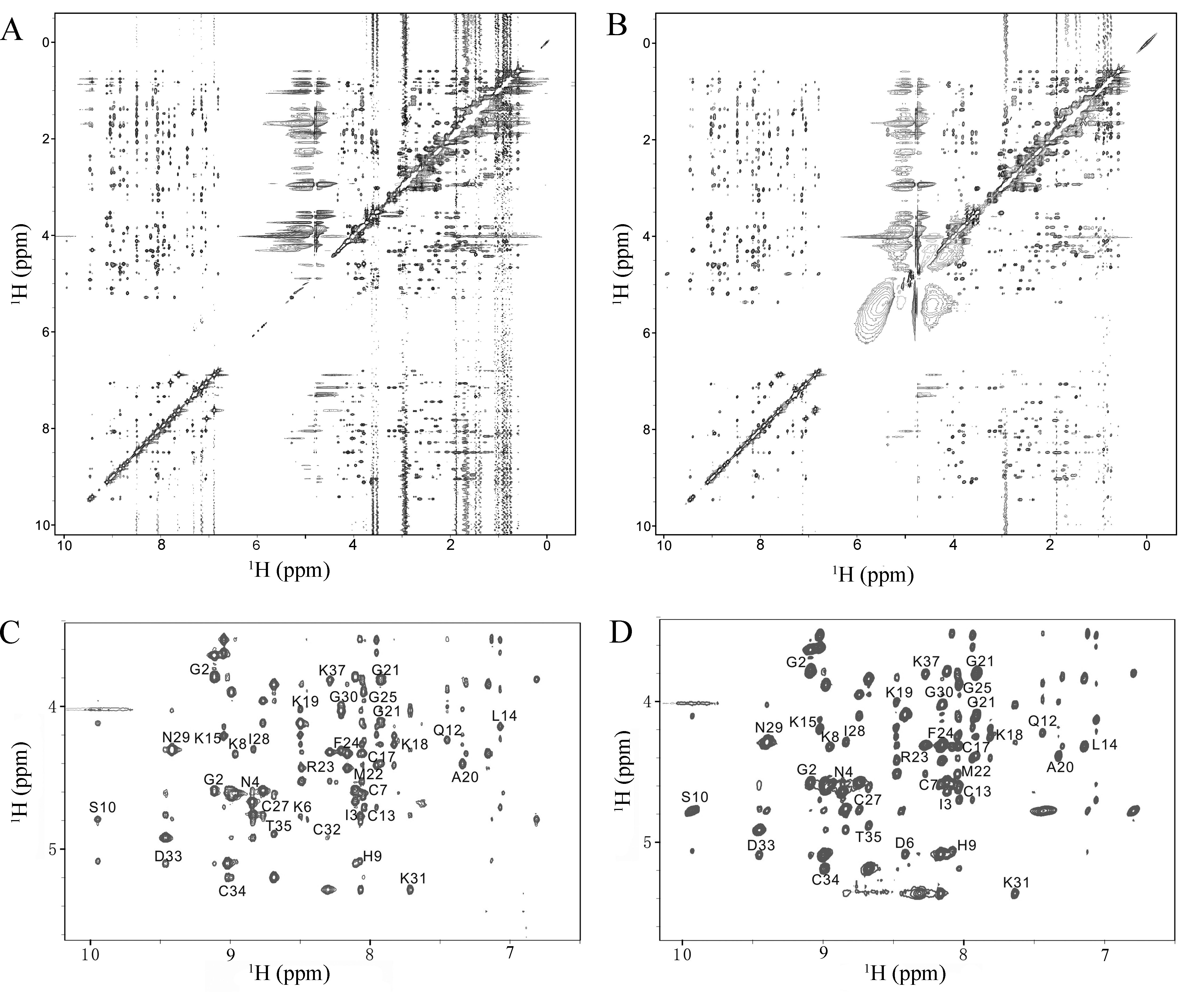


Figure S3. 2D ^1^H-^1^H NOESY spectra of BmKTX-19 and BmKTX-196. NOESY spectra of BmKTX-19 (A) and BmKTX-196 (C); The HN-HA regions of BmKTX-19 (B) and BmKTX-196 (D) labeled by the residue name and number.


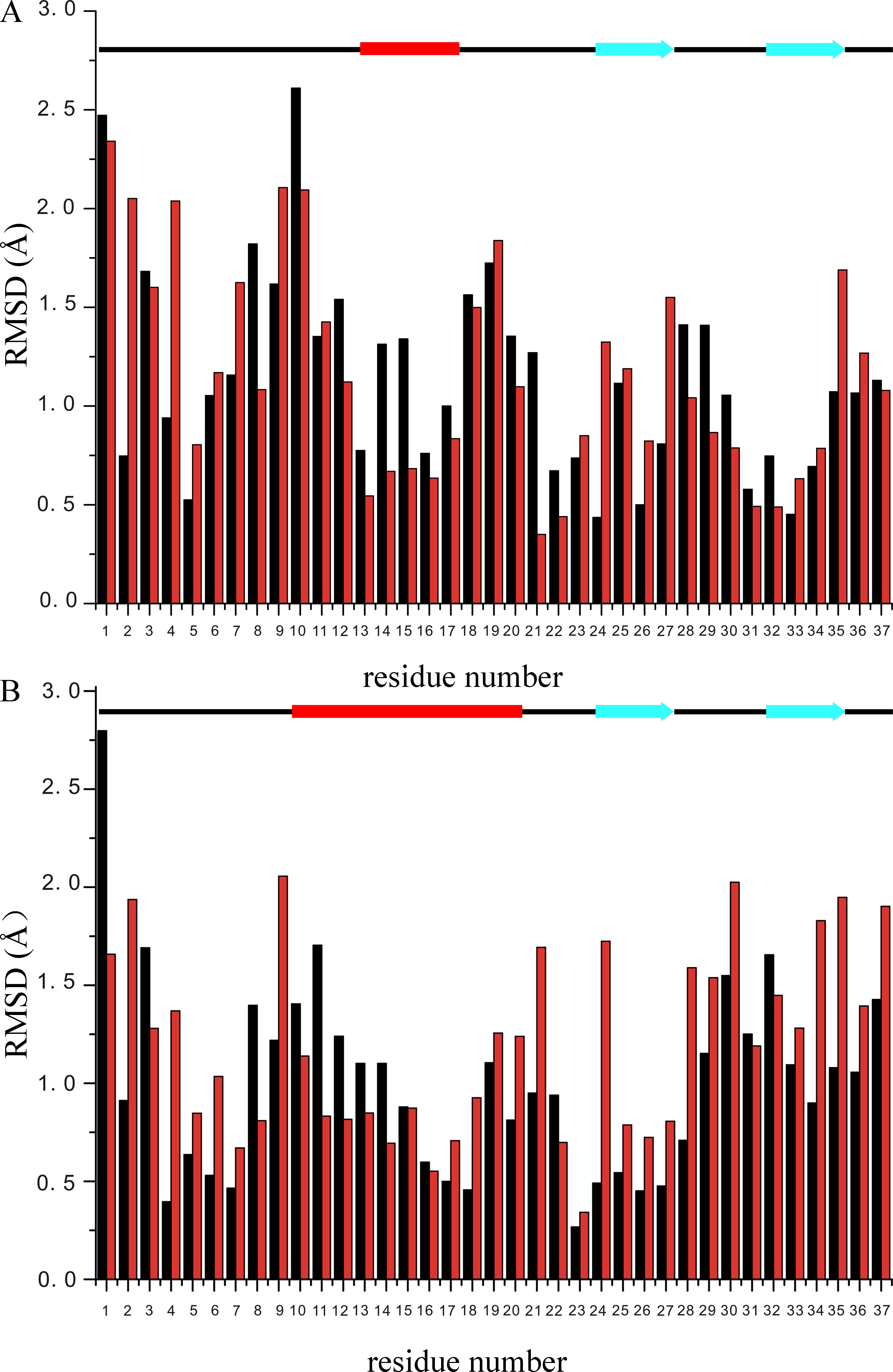


Figure S4. The plot of the backbone RMSD versus the residue number. A, RMSD between BmKTX-19 and wild-type BmKTX (black), and ADWX-1 (red). B, RMSD between BmKTX-196 and wild-type BmKTX (black), and ADWX-1 (red).


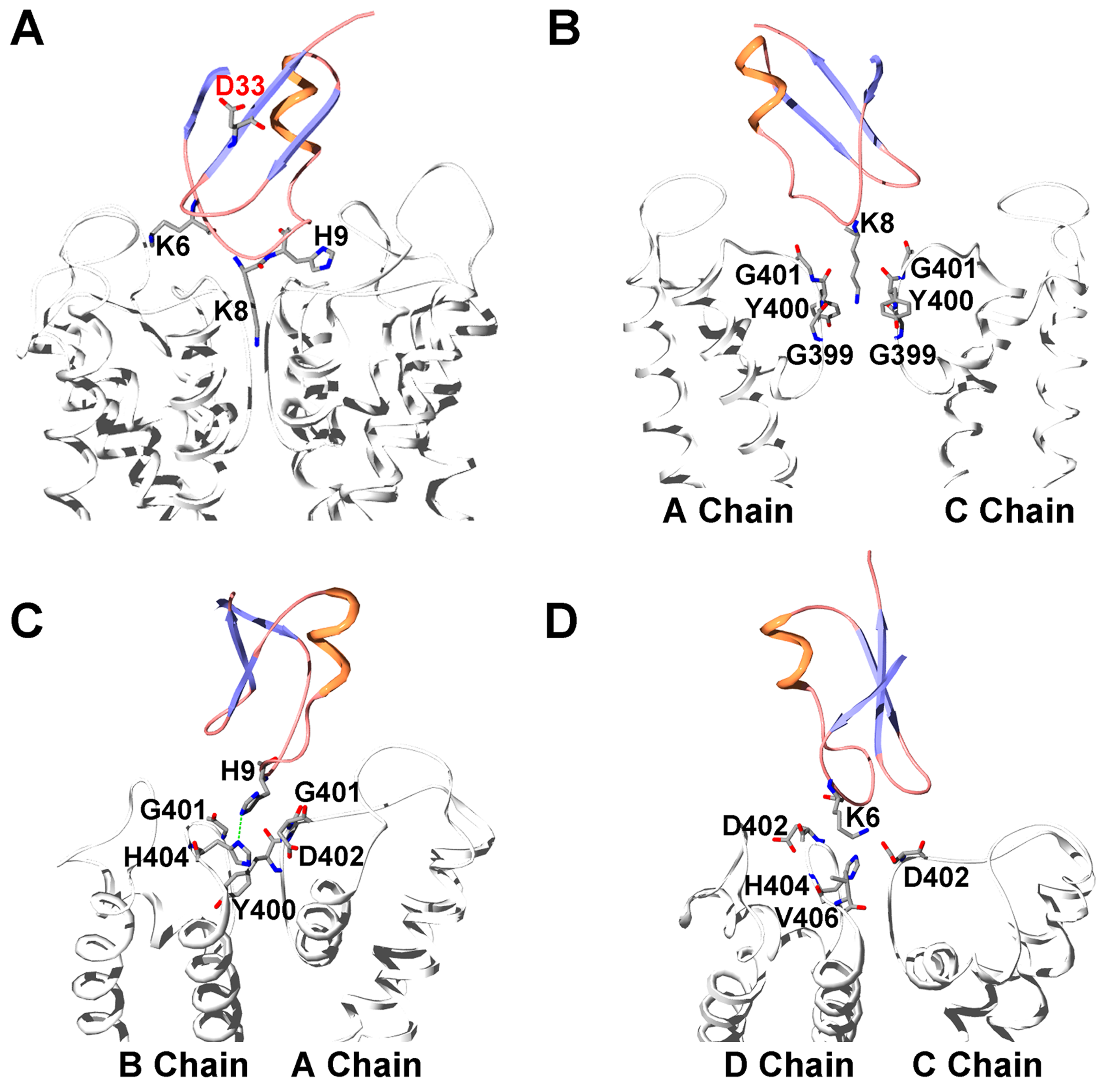


Figure S5. Interaction modes of BmKTX-19 peptides with Kv1.3 channels. A, Interaction mode of BmKTX-19 with Kv1.3 channel. BmKTX-19 acidic residues and key functional residues were marked. B, Kv1.3 channel pore-blocking Lys8 of BmKTX-19 mainly contacted the conserved residues of Kv1.3 channel pore region within a contact distance of 4 Å. C, His9 of BmKTX-19 interacted with Tyr400, Gly401 and Asp402 in channel A chain, Gly401 and His404 in channel B chain within a contact distance of 4 Å. D, Lys6 of BmKTX-19 mainly contacted channel Asp402 in C chian, Asp402, His404 and Val406 in D chain within a contact distance of 4 Å.


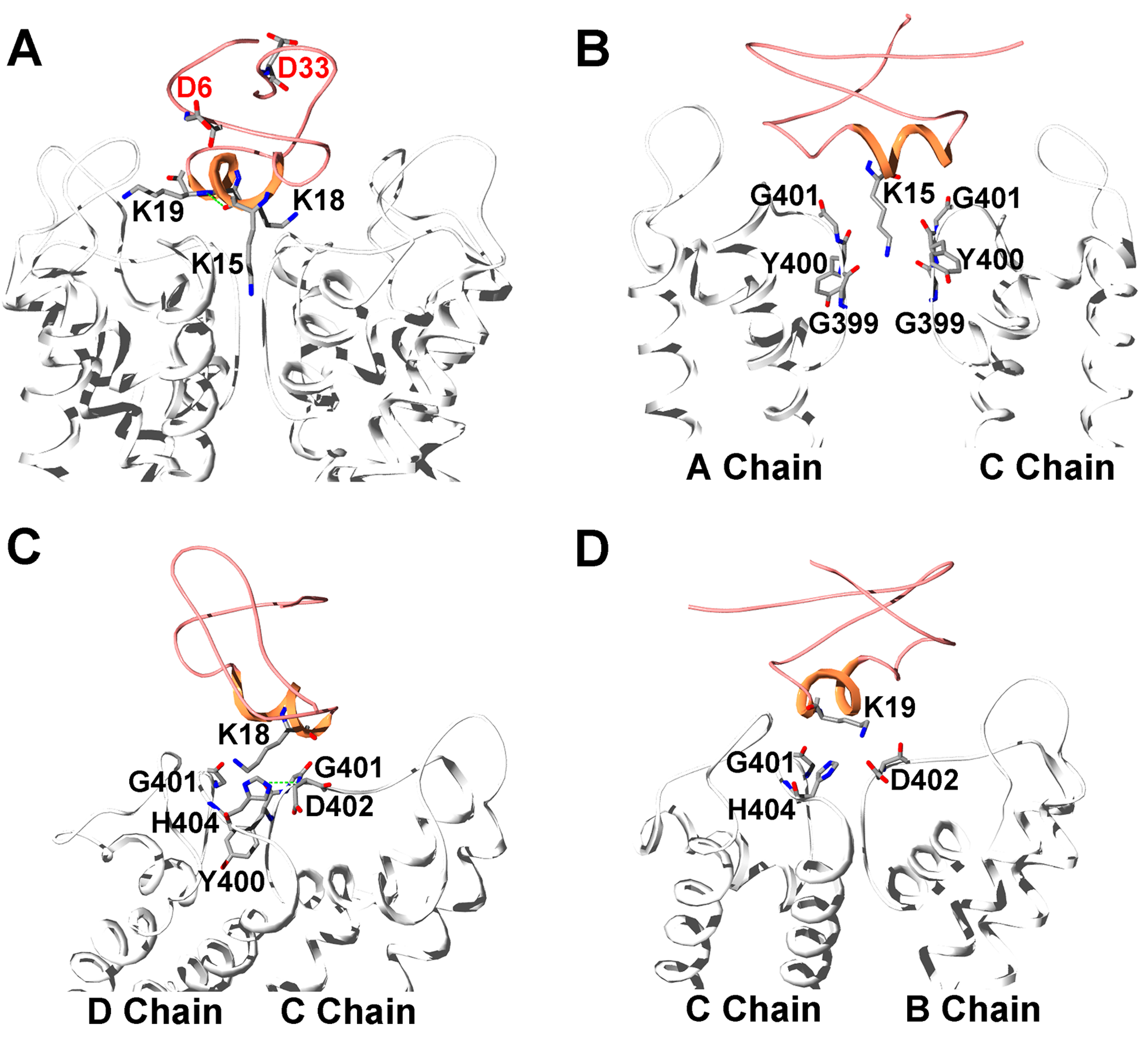


Figure S6. Interaction modes of BmKTX-196 peptides with Kv1.3 channels. A, Interaction mode of BmKTX-196 with Kv1.3 channel. BmKTX-196 acidic residues and key functional residues were marked. B, Kv1.3 channel pore-blocking Lys15 of BmKTX-196 was surrounded by residues from Kv1.3 pore and turret regions within a contact distance of 4 Å. C, Lys18 of BmKTX-196 interacted with Tyr400, Gly401 and Asp402 in channel C chain, Gly401 and His404 in channel D chain within a contact distance of 4 Å. D, Lys19 of BmKTX-196 mainly interacted with Asp402 in channel B chain, Gly401 and His404 in channel C chain within a contact distance of 4 Å.
